# Efficacy and safety of ozone autohemotherapy for zoster-associated pain: a meta-analysis and trial sequential analysis

**DOI:** 10.3389/fneur.2026.1812211

**Published:** 2026-06-03

**Authors:** Chunmei Wu, Zehao Liu, Yixin Zhang, Yangyang Li, Funing Liu, Yong Chen, Xiaojuan Liu, An Yang, Huide Wang, Qing Zhong

**Affiliations:** 1 Department of Anesthesiology and Pain Medicine, The People's Hospital of Jianyang, Jianyang, China; 2 Department of Anesthesiology, The Affiliated Hospital of Southwest Medical University, Luzhou, China; 3 Department of Science and Technology, The People's Hospital of Jianyang, Jianyang, China

**Keywords:** meta-analysis, ozone autohemotherapy, treatment, trial sequential analysis, zoster-associated pain

## Abstract

**Objective:**

To systematically evaluate the efficacy and safety of ozone autohemotherapy (O₃-AHT) in the treatment of zoster-associated pain (ZAP).

**Methods:**

PubMed, Cochrane Library, Web of Science, Embase, Chinese Biomedical Database, China National Knowledge Infrastructure (CNKI), Wanfang Database, and VIP Database were searched on September 10, 2025. We searched for randomized controlled trials evaluating O₃-AHT for ZAP management from database inception to September 10, 2025. Two researchers independently screened the literature, extracted data, and assessed the risk of bias. Data analysis was performed using RevMan 5.4 and Stata 18.0 software to calculate the standardized mean difference (SMD), mean difference (MD), relative risk (RR), and their 95% confidence intervals (CI). Trial sequential analysis (TSA) was conducted using TSA 0.9.5.10 software to assess the robustness of the evidence, and the Grading of Recommendations Assessment, Development and Evaluation (GRADE) system was used to evaluate the quality of evidence.

**Results:**

A total of 20 studies involving 1,519 patients were included. Meta-analysis revealed that compared with the control group, O_3_-AHT significantly patient self-reported pain scores (visual analog scale [VAS] or numerical rating scale [NRS]) compared to controls (SMD = −1.77, 95% CI: −2.16 to −1.37, *p* < 0.01), improved the effective rate of pain relief (RR = 1.21, 95% CI: 1.09 to 1.33, *p* < 0.01), decreased inflammatory factor levels (e.g., IL-6: SMD = −1.84, 95% CI: −2.60 to −1.07, *p* < 0.01), and improved quality of life scores (MD = 0.75, 95% CI: 0.45 to 1.04, *p* < 0.01). No significant difference was observed in the incidence of adverse reactions between the two groups (RR = 0.77, 95% CI: 0.46 to 1.28, *p* = 0.31). However, substantial heterogeneity existed among studies (I^2^ often > 50%). TSA suggested that the current cumulative sample size exceeded the required information size for primary outcome (pain score), thereby supporting the reliability of the conclusion. GRADE evidence quality rating was very low to moderate, mainly limited by the risk of bias and heterogeneity of included studies.

**Conclusion:**

O₃-AHT may effectively alleviate ZAP and improve the quality of life and sleep quality through anti-inflammatory and immunomodulatory mechanisms, with a favorable safety profile; moreover, combination therapy may offer greater clinical benefit. However, owing to limitations in the number and quality of included studies, further larger-scale, high-quality randomized controlled trials are warranted.

**Systematic review registration:**

https://www.crd.york.ac.uk/PROSPERO/, identifier: CRD420251145896.

## 1 Introduction

Zoster-associated pain (ZAP) is a neuropathic pain condition caused by reactivation of the varicella-zoster virus, characterized by severe and persistent pain, including burning sensations, electric shock-like pain, and abnormal mechanical hypersensitivity. This pain, which may persist even after healing of the herpes zoster rash, can transform into a chronic pain syndrome, severely affecting patients’ sleep quality, psychological state, and daily functioning (1). Epidemiological studies have demonstrated that approximately 20% of patients with herpes zoster develop postherpetic neuralgia (PHN) (2), with nearly 80% of cases occurring in individuals aged 50 and above. With the acceleration of global population aging, the disease burden of ZAP is expected to further increase, posing substantial challenges to healthcare systems (3).

Current clinical treatment approaches for ZAP primarily include pharmacological therapy (such as anticonvulsants, tricyclic antidepressants, and opioids) (4) and interventional therapy (5) (such as pulsed radiofrequency [PRF] and nerve blocks). However, the effectiveness of pharmacological treatment is often unsatisfactory (6), and older patients using these medications may face issues with adverse effects and long-term dependence. In recent years, ozone autohemotherapy (O₃-AHT), as an effective adjuvant or combination therapy approach, has demonstrated broad application prospects in the field of pain management (7–9). Ozone (O₃) is an allotrope of oxygen with strong oxidative properties that can exert therapeutic effects through multiple mechanisms (10): inhibiting the release of pain-induced inflammatory factors in peripheral tissues (such as serotonin and bradykinin); improving local tissue metabolism and alleviating hypoxic conditions at nerve endings; and enhancing anti-inflammatory cytokine activity by stimulating the release of endogenous antioxidant enzymes.

Although several clinical studies have explored the effects of O₃-AHT in the treatment of ZAP, the existing evidence mostly comes from single-center trials with limited sample sizes and varying methodological quality, resulting in inconsistent results and insufficient statistical efficacy, making it difficult to form reliable conclusions (11–19). Both O₃-AHT monotherapy or combination therapy can significantly improve the treatment success rate for ZAP (20, 21). However, these studies are mostly single-center, small-sample trials, and there is an urgent need to synthesize existing evidence through systematic review and meta-analysis methods to provide more reliable evidence for clinical practice.

Therefore, to address this evidence gap, we conducted a systematic review and meta-analysis to comprehensively search for randomized controlled trials (RCTs) on O₃-AHT for the treatment of ZAP, evaluating its efficacy and safety, thereby providing high-level, evidence-based support for clinical decision-making.

## 2 Methods

### 2.1 Study design and registration

This study protocol has been registered with the PROSPERO International Prospective Register of Systematic Reviews (Registration Number: CRD420251145896) and this study strictly follows the Preferred Reporting Items for Systematic Reviews (PRISMA) and Meta - Analyses 2020 statement to report (22, 23).

### 2.2 Literature search strategy

We searched PubMed, Embase, Cochrane Library, Web of Science, China National Knowledge Infrastructure (CNKI) and Wanfang Database, Chinese BioMedical Database, and VIP Database to collect relevant literature from database inception to September 10, 2025. Through the Medical Subject Headings (MeSH) thesaurus, we identified keywords and their Chinese equivalents. The search strategy combined MeSH terms with free-text terms and was tailored to the characteristics of each database. When necessary, reference lists of included studies were manually searched to supplement the retrieval of relevant literature. This systematic review did not actively search for grey literature (e.g., unpublished trials, conference abstracts) because its methodological quality is often uneven and may introduce additional bias. During the preparation of the revised version, we conducted a supplementary search and found no new studies that met the inclusion criteria (Table 1).

**Table 1:** Database search strategy.

Databases	Search strategy
PubMed	(((((((((((ozone[MeSH Terms]) OR (ozone[Title/Abstract])) OR (triplet oxygen[Title/Abstract])) OR (oxygen, triplet[Title/Abstract])) OR (ozonated[Title/Abstract])) OR (O3[Title/Abstract])) OR (ozonated autohemotherapy[Title/Abstract])) OR (ozone autohemotherapy[Title/Abstract])) OR (intravenous ozone therapy[Title/Abstract])) OR (major autohemotherapy[Title/Abstract])) AND ((((((((((((herpes zoster[MeSH Terms]) OR (herpes zoster[Title/Abstract])) OR (zoster, herpes[Title/Abstract])) OR (herpes zona[Title/Abstract])) OR (zoster[Title/Abstract])) OR (zona[Title/Abstract])) OR (shingles[Title/Abstract])) OR (herpes zoster-related neuralgia[Title/Abstract])) OR (zoster-related neuralgia[Title/Abstract])) OR (neuralgia, postherpetic[MeSH Terms])) OR (neuralgia, postherpetic[Title/Abstract])) OR (postherpetic neuralgia[Title/Abstract]))) AND (((((((((((random allocation[MeSH Terms]) OR (random allocation[Title/Abstract])) OR (allocation, random[Title/Abstract])) OR (randomized controlled trial[Title/Abstract])) OR (randomized controlled trials[Title/Abstract])) OR (random[Title/Abstract])) OR (randomized[Title/Abstract])) OR (randomised[Title/Abstract])) OR (randomly[Title/Abstract])) OR (randomization[Title/Abstract])) OR (RCT[Title/Abstract]))
Cochrane Library	#1 MeSH descriptor: [Ozone] explode all trees #2 (ozone):ti,ab,kw OR (triplet oxygen):ti,ab,kw OR (oxygen, triplet):ti,ab,kw OR (ozonated):ti,ab,kw OR (O3):ti,ab,kw OR (ozonated autohemotherapy):ti,ab,kw OR (ozone autohemotherapy):ti,ab,kw OR (major autohemotherapy):ti,ab,kw OR (intravenous ozone therapy):ti,ab,kw #3 #1 OR #2 #4 MeSH descriptor: [Herpes Zoster] explode all trees #5 MeSH descriptor: [Neuralgia, Postherpetic] explode all trees #6 (herpes zoster):ti,ab,kw OR (zoster, herpes):ti,ab,kw OR (herpes zona):ti,ab,kw OR (zoster):ti,ab,kw OR (zona):ti,ab,kw OR (shingles):ti,ab,kw OR (herpes zoster-related neuralgia):ti,ab,kw OR (zoster-related neuralgia):ti,ab,kw OR (neuralgia, postherpetic):ti,ab,kw OR (postherpetic neuralgia):ti,ab,kw #7 #4 OR #5 OR #6 #8 MeSH descriptor: [Random Allocation] explode all trees #9 (random allocation):ti,ab,kw OR (allocation, random):ti,ab,kw OR (randomized controlled trial):ti,ab,kw OR (randomized controlled trials):ti,ab,kw OR (random):ti,ab,kw OR (randomized):ti,ab,kw OR (randomised):ti,ab,kw OR (randomly):ti,ab,kw OR (randomization):ti,ab,kw OR (RCT):ti,ab,kw #10 #8 OR #9 #11 #3 AND #7 AND #10
Web of Science	#1: TS = (ozone) OR TS = (triplet oxygen) OR TS = (oxygen, triplet) OR TS = (ozonated) OR TS = (O3) OR TS = (ozonated autohemotherapy) OR TS = (ozone autohemotherapy) OR TS = (major autohemotherapy) OR TS = (intravenous ozone therapy) #2: TS = (herpes zoster) OR TS = (zoster, herpes) OR TS = (herpes zona) OR TS = (zoster) OR TS = (zona) OR TS = (shingles) OR TS = (herpes zoster-related neuralgia) OR TS = (zoster-related neuralgia) OR TS = (neuralgia, postherpetic) OR TS = (postherpetic neuralgia) #3: TS = (random allocation) OR TS = (allocation, random) OR TS = (randomized controlled trial) OR TS = (randomized controlled trials) OR TS = (random) OR TS = (randomized) OR TS = (randomised) OR TS = (randomly) OR TS = (randomization) OR TS = (RCT) #4: #1 AND #2 AND #3
Embase	#3 AND #7 AND #10 #10: #8 OR #9 #9: ‘random allocation’:ti,ab,kw OR ‘allocation, random’:ti,ab,kw OR ‘randomized controlled trial’:ti,ab,kw OR ‘randomized controlled trials’:ti,ab,kw OR ‘random’:ti,ab,kw OR ‘randomized’:ti,ab,kw OR ‘randomised’:ti,ab,kw OR ‘randomly’:ti,ab,kw OR ‘randomization’:ti,ab,kw OR ‘rct’:ti,ab,kw #8: ‘randomization’/exp. OR ‘randomization’ #7: #4 OR #5 OR #6 #6: ‘herpes zoster’:ti,ab,kw OR ‘zoster, herpes’:ti,ab,kw OR ‘herpes zona’:ti,ab,kw OR ‘zoster’:ti,ab,kw OR ‘zona’:ti,ab,kw OR ‘shingles’:ti,ab,kw OR ‘herpes zoster-related neuralgia’:ti,ab,kw OR ‘zoster-related neuralgia’:ti,ab,kw OR ‘neuralgia, postherpetic’:ti,ab,kw OR ‘postherpetic neuralgia’:ti,ab,kw #5: ‘postherpetic neuralgia’/exp. OR ‘postherpetic neuralgia’ #4: ‘herpes zoster’/exp. OR ‘herpes zoster’ #3: #1 OR #2 #2: ‘ozone’:ti,ab,kw OR ‘triplet oxygen’:ti,ab,kw OR ‘oxygen, triplet’:ti,ab,kw OR ‘ozonated’:ti,ab,kw OR ‘o3’:ti,ab,kw OR ‘ozonated autohemotherapy’:ti,ab,kw OR ‘ozone autohemotherapy’:ti,ab,kw OR ‘major autohemotherapy’:ti,ab,kw OR ‘intravenous ozone therapy’:ti,ab,kw #1: ‘ozone’/exp. OR ‘ozone’
CBM-disc	(“臭氧”[常用字段:智能] OR “三氧”[常用字段:智能] OR “O3”[常用字段:智能] OR “臭氧自体血回输”[常用字段:智能] OR “三氧自体血回输”[常用字段:智能] OR “臭氧自血回输”[常用字段:智能] OR “三氧自血回输”[常用字段:智能] OR “臭氧大自血”[常用字段:智能] OR “三氧大自血”[常用字段:智能]) AND (“带状疱疹”[常用字段:智能] OR “蛇丹”[常用字段:智能] OR “蛇串疮”[常用字段:智能] OR “火带疮”[常用字段:智能] OR “蜘蛛疮”[常用字段:智能] OR “带状疱疹疼痛”[常用字段:智能] OR “带状疱疹神经痛”[常用字段:智能] OR “带状疱疹相关神经痛”[常用字段:智能] OR “神经痛, 带状疱疹后”[常用字段:智能] OR “带状疱疹后神经痛”[常用字段:智能] OR “带状疱疹后遗神经痛”[常用字段:智能]) AND (“随机分配”[常用字段:智能] OR “随机对照实验”[常用字段:智能] OR “随机对照”[常用字段:智能] OR “随机”[常用字段:智能] OR “随机化”[常用字段:智能] OR “随机分组”[常用字段:智能] OR “RCT”[常用字段:智能])
CNKI	(篇关摘:带状疱疹后神经痛(模糊)) AND (篇关摘:脊髓电刺激(模糊)) AND (篇关摘:脉冲射频(模糊)) Translation: (Abstract: Postherpetic Neuralgia (Fuzzy)) AND (Abstract: Spinal Cord Stimulation (Fuzzy)) AND (Abstract: Pulsed Radiofrequency (Fuzzy))
Wanfang	题名或关键词:(带状疱疹后神经痛) AND 题名或关键词:(脊髓电刺激) AND 题名或关键词:(脉冲射频) Translation: Title or keywords: (Postherpetic Neuralgia) AND Title or keywords: (Spinal Cord Stimulation) AND Title or keywords: (Pulsed Radiofrequency)
VIP	(主题:(“臭氧”) or 摘要:(“臭氧” or “三氧” or “O3” or “臭氧自体血回输” or “三氧自体血回输” or “臭氧自血回输” or “三氧自血回输” or “臭氧大自血” or “三氧大自血”)) and (主题:(“带状疱疹”) or 主题:(“神经痛, 带状疱疹后”) or 摘要:(“带状疱疹” or “蛇丹” or “蛇串疮” or “火带疮” or “蜘蛛疮” or “带状疱疹疼痛” or “带状疱疹神经痛” or “带状疱疹相关神经痛” or “神经痛, 带状疱疹后” or “带状疱疹后神经痛” or “带状疱疹后遗神经痛”)) and (主题:(“随机分配”) or 摘要:(“随机分配” or “随机对照实验” or “随机对照” or “随机” or “随机化” or “随机分组” or “RCT”))

### 2.3 Inclusion and exclusion criteria

Inclusion criteria: ① Study type: RCTs; ② Study participants: patients meeting the diagnostic criteria for herpes zoster neuralgia; ③ Intervention: the experimental group received O₃-AHT, either alone or in combination with conventional treatments; ④ Control group: received conventional pharmacological treatment, placebo, or other non-ozone-based therapies; ⑤ Outcome measures: including at least one of the following indicators: visual analog scale (VAS) or numerical rating scale (NRS), Pittsburgh Sleep Quality Index (PSQI), World Health Organization Quality of Life-BREF (WHOQOL-BREF), response rate (decrease in patient-reported pain score > 50% or patient-reported pain score < 5), inflammatory factors (interleukin IL-6, IL-1β, tumor necrosis factor TNF-*α*), immune function indicators (CD4^+^/CD8^+^ ratio).

Exclusion criteria: ① Non-randomized studies, retrospective studies, case reports, reviews, and conference abstracts; ② Studies with incomplete data or unavailable full text; ③ Duplicate publications; and ④ the research design was unreasonable and studies published in languages other than Chinese and English.

### 2.4 Data extraction and quality assessment

Two researchers independently screened the literature, extracted data, and cross-validated the results. In case of disagreements, a third researcher was consulted or the issue was resolved through discussion. The data extraction included: ① Basic study information: first author and publication year; ② Study characteristics: sample size, age, disease duration, details of intervention measures, control measures, and follow-up time; and ③ Outcome indicators: primary outcome (pain scores) and secondary outcomes (pain relief effectiveness rate, inflammatory factor levels, immune function indicators, sleep quality and quality of life scores, and adverse reaction incidence rate).

The risk of bias in included studies was assessed using the Cochrane Risk of Bias tool, which covers the following seven domains: random sequence generation, allocation concealment, blinding of participants and personnel, blinding of outcome assessment, completeness of outcome data, selective reporting, and other biases. Each domain was rated as “low risk,” “high risk,” or “unclear”.

### 2.5 Statistical analysis

Data analysis was performed using RevMan 5.4 and Stata 18.0 software. When the outcome index was dichotomous data, quantitative analysis used relative risk (RR) and 95% confidence interval (CI). When the outcome index was continuous data, quantitative analysis used mean difference (MD) and 95% confidence interval. If the indicator measurement methods differed, standardized mean difference (SMD) and 95% CI were used for quantitative analysis. We believe that a reduction in the patient’s self-reported pain score ≥1.5 points or ≥ 30% is clinically significant (24, 25).

Heterogeneity analysis used Higgins I^2^ statistic and Cochrane’s Q test. If I^2^ ≥ 50% or *p* < 0.1 for the Q test, the heterogeneity was considered large, and a random-effects model was used for analysis. Otherwise, a fixed-effects model was used for analysis. For results with significant heterogeneity, subgroup analysis was employed to determine the source of heterogeneity. Additionally, we explored potential sources of heterogeneity. To assess the robustness of the findings, we performed a series of sensitivity analyses by sequentially excluding individual studies and examining their impact on the overall results. Egger’s Test and Begg’s Test were used to detect publication bias. When the number of included studies was limited, subgroup, sensitivity, and publication bias analyses were not performed. Differences were considered statistically significant at *p* < 0.05.

For primary outcome measure (pain scores), we will perform trial sequential analyses (TSA) using TSA 0.9.5.10 software to assess whether the current cumulative evidence is sufficient to avoid random error. The preset type I error (*α*) is 5%, the test power (1-*β*) is 80%, and the required amount of information is calculated based on the observed effect size.

The quality of the evidence for the primary outcome was assessed using the Grading of Recommendations Assessment, Development and Evaluation (GRADE) system. The level of evidence will start at “high” and be downgraded based on five dimensions: risk of bias, inconsistency, indirectness, imprecision and publication bias.

## 3 Results

### 3.1 Literature screening process and results

**Figure 1:**
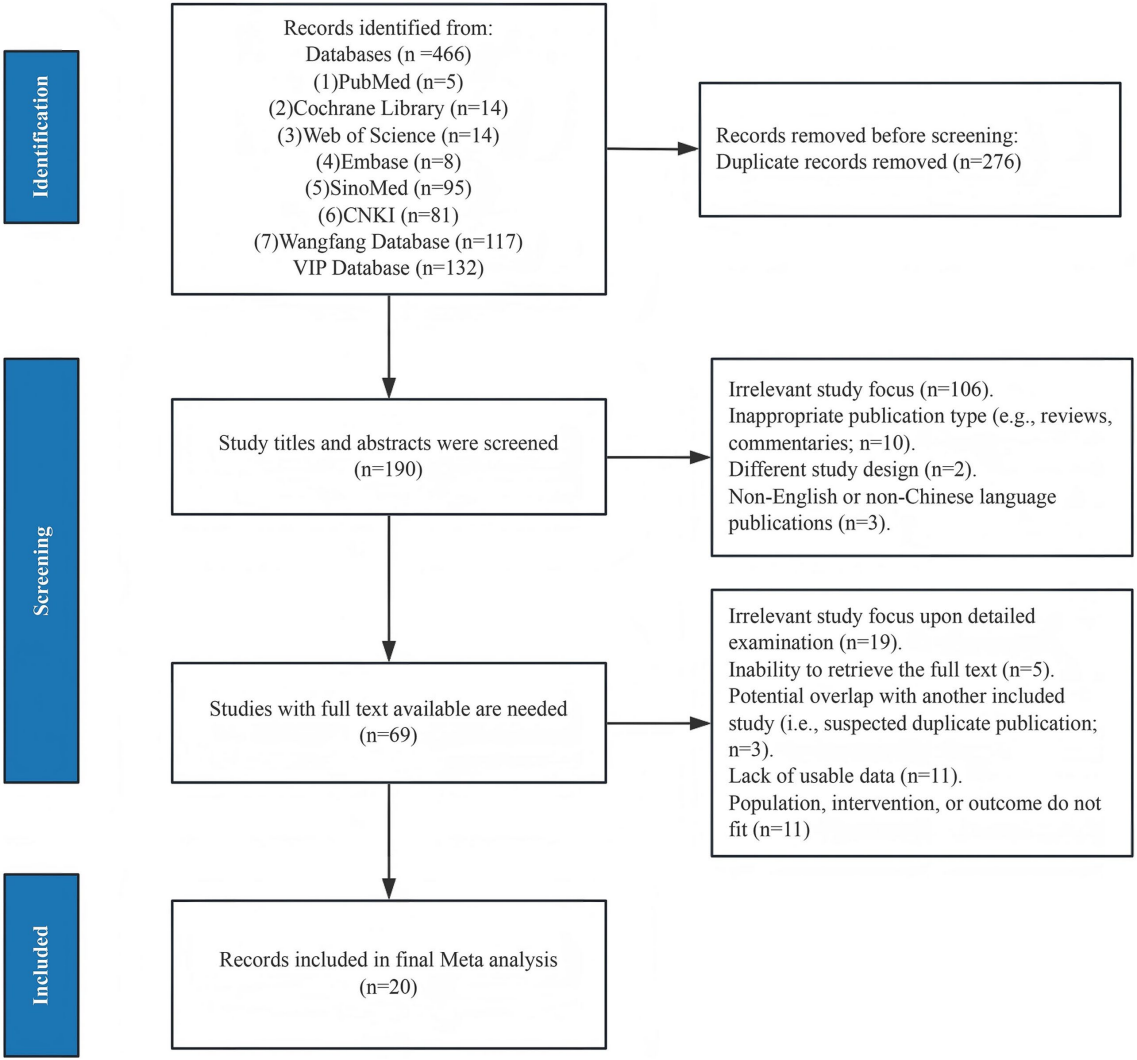
PRISMA flowchart of literature screening.

A total of 466 relevant articles were initially retrieved. After stepwise screening, 20 RCTs (12–19, 26–37) involving a total of 1,519 patients were finally included. The literature screening process and results are shown in the PRISMA flowchart (Figure 1).

### 3.2 Basic characteristics of included studies

The 20 included studies were published from 30 January 2017 to 10 September 2025, with sample sizes ranging from 20 to 104 cases. Ozone therapy modalities included O_3_-OAH + drug+nerve block (4 studies) (15, 16, 26, 35), O_3_-OAH + drug (14 studies) (12, 14, 18, 19, 27–34, 36, 37), O_3_-OAH + PRF (1 study) (17), O_3_-OAH + drug+ high-voltage PRF [HVPRF] (1 study) (13). Control group interventions included conventional pharmacological therapy (pregabalin and gabapentin), PRF therapy, and nerve block procedures. Nineteen studies reported patient-reported pain scores,5 studies reported pain relief efficacy rates, 6 studies reported the TNF-*α* levels, 6 studies reported the IL-6 levels, 2 studies reported the IL-1β levels, 4 studies reported the CD4^+^/CD8^+^ levels, 2 studies reported the quality of life scores, 2 studies reported sleep quality outcomes, and 8 studies reported adverse events (Table 2).

**Table 2:** Basic characteristics of included studies.

Authors	Year	Sample size (T/C)	Intervention	Comparison	O3-OAH	Available outcomes	Follow-up points
Hua et al (17)	2018	20/20	O3-OAH + Drug+Nerve Block	Drug + Nerve Block	Concentration: 35ug/ml Volume: 100 mL Course of treatment: qd, 10d	VAS	7D, 14D, 28D
Zheng et al (35)	2025	53/40	O3-OAH + Drug	Drug	Concentration: 30ug/ml Volume: 50 mL/time*3 Course of treatment: bid 15d	VAS adverse reaction	7D, 15D
Hu et al (34)	2018	46/45	O3-OAH + Drug	Drug	Concentration: 30ug/ml Volume: 40 mL Course of treatment: 3 times /7d, 14d	VAS WHOQOL-BREF	1 W, 1 M, 3 M
Wei et al (36)	2019	65/60	O3-OAH + Drug	Drug	Concentration: First time: 25ug/ml; 2nd: 30ug/ml; 3re: 35ug/ml; ≥4th: 40ug/ml Volume: 200 mL Course of treatment: qd, 7d	VAS CD4+/CD8+ IL-6 TNF-*α* adverse reaction	1D, 3D, 7D
Wu et al (27)	2024	60/60	O3-OAH + PRF	PRF	Concentration: - Volume: 100 mL Course of treatment: qd, 20d	VAS CD4+/CD8+ adverse reaction	1D, 3D, 7D
Wu et al (28)	2022	20/20	O3-OAH + Drug	Drug	Concentration: 25ug/ml Volume: 100 mL Course of treatment: qd, 5d	VAS CD4+/CD8+	4D, 6D
Zhang et al (29)	2020	40/40	O3-OAH + Drug	Drug	Concentration: 37–40 μg/mL Volume: 100 mL Course of treatment: qd, 15d	VAS adverse reaction	3 M
Chao et al (14)	2023	31/31	O3-OAH + Drug	Drug	Concentration:30-50ug/ml Volume: 100 mL Course of treatment:1time/3d, 30d	VAS IL-6 TNF-α	10D, 20D, 30D
Zhu et al (30)	2017	50/50	O3-OAH + Drug+Nerve Block	Drug + Nerve Block	Concentration: 47ug/ml Volume: 100 ml Course of treatment: qd, 7d	VAS	1 W, 2 W, 3 W, 4 W, 6 W, 8 W, 12 W
Li et al (19)	2023	30/30	O3-OAH + Drug+Nerve Block	Drug + Nerve Block	Concentration: 25ug/ml Volume: 100 ml Course of treatment: qd, 7d	VAS IL-6 TNF-α adverse reaction	7D
Li et al (20)	2018	44/44	O3-OAH + Drug	Drug	Concentration: First time: 20ug/ml; 2nd: 25ug/ml; 3rd: 30ug/ml; ≥4th: 40ug/ml Volume: 100 ml Course of treatment: qd, 10d*3	VAS	10D, 25D, 40D
Tu et al (24)	2018	28/28	O3-OAH + Drug	Drug	Concentration:30ug/ml Volume: 40 mL Course of treatment: qd, 20d	VAS	1 W, 1 M, 3 M
Wang et al (26)	2022	25/23	O3-OAH + Drug/+subcutaneous injection	Drug/+subcutaneous injection	Concentration: First time: 20ug/ml; 2nd: 25ug/ml; 3rd: 30ug/ml; ≥4th: 40ug/ml Volume: 150 mL Course of treatment: qd, 10d	VAS adverse reaction	3D, 7D, 15D, 1 M, 3 M, 6 M
Wang et al (25)	2023	20/20	O3-OAH + Drug	Drug	Concentration: First time: 25ug/ml; 2nd: 30ug/ml; 3rd: 35ug/ml; ≥4th: 40ug/ml Volume: 100 mL Course of treatment: qd, 14d	VAS CD4+/CD8+ adverse reaction	8D, 15D
Su et al (23)	2021	24/23	O3-OAH + Drug	Drug	Concentration: - Volume: 100 ml Course of treatment: qd, 14d	VAS PSQI IL-6 TNF-α	30D
Mo et al (22)	2021	51/52	O3-OAH + Drug	Drug	Concentration: 40ug/ml Volume: 100 ml Course of treatment:3 times /7d, 30d	VAS WHOQOL-BREF IL-1β TNF-α	1 W, 2 W, 1 M
Jia et al (18)	2023	30/30	O3-OAH + Drug+Nerve Block	Drug + Nerve Block	Concentration:25ug/ml Volume: 100 ml Course of treatment: qd, 7d	VAS IL-6 IL-1β	VAS:7D IL-6 and IL-1β:4D, 7D
Guo et al (16)	2021	39/39	O3-OAH + Drug	Drug	Concentration: First time: 25ug/ml; 2nd: 30ug/ml; ≥3rd: 40ug/ml Volume: 100 ml Course of treatment: qd, 10d	VAS	5D, 10D, 20D, 30D
Chen et al (15)	2024	45/45	O3-OAH + Drug+HVPRF	Drug + HVPRF	Concentration: First time: 20ug/ml; 2nd: 25ug/ml; 3rd: 30ug/ml; ≥4th: 40ug/ml Volume: 100 ml Course of treatment: qd, 6d	VAS PSQI IL-6 TNF-α adverse reaction	7D, 1 M, 3 M, 6 M
Ma et al (21)	2019	50/50	O3-OAH + Drug	Drug	Concentration: 0-60ug/ml Volume: - Course of treatment: qd, 15d	NRS	after treatment

T/C: test group/control group; O_3_ –AHT, ozone autohemotherapy; PRF, pulsed radiofrequency; HVPRF, high voltage pulsed radiofrequency. D=Day, W=Week, M = Month.

### 3.3 Meta-analysis results

#### 3.3.1 Patient self-reported pain score

**Figure 2:**
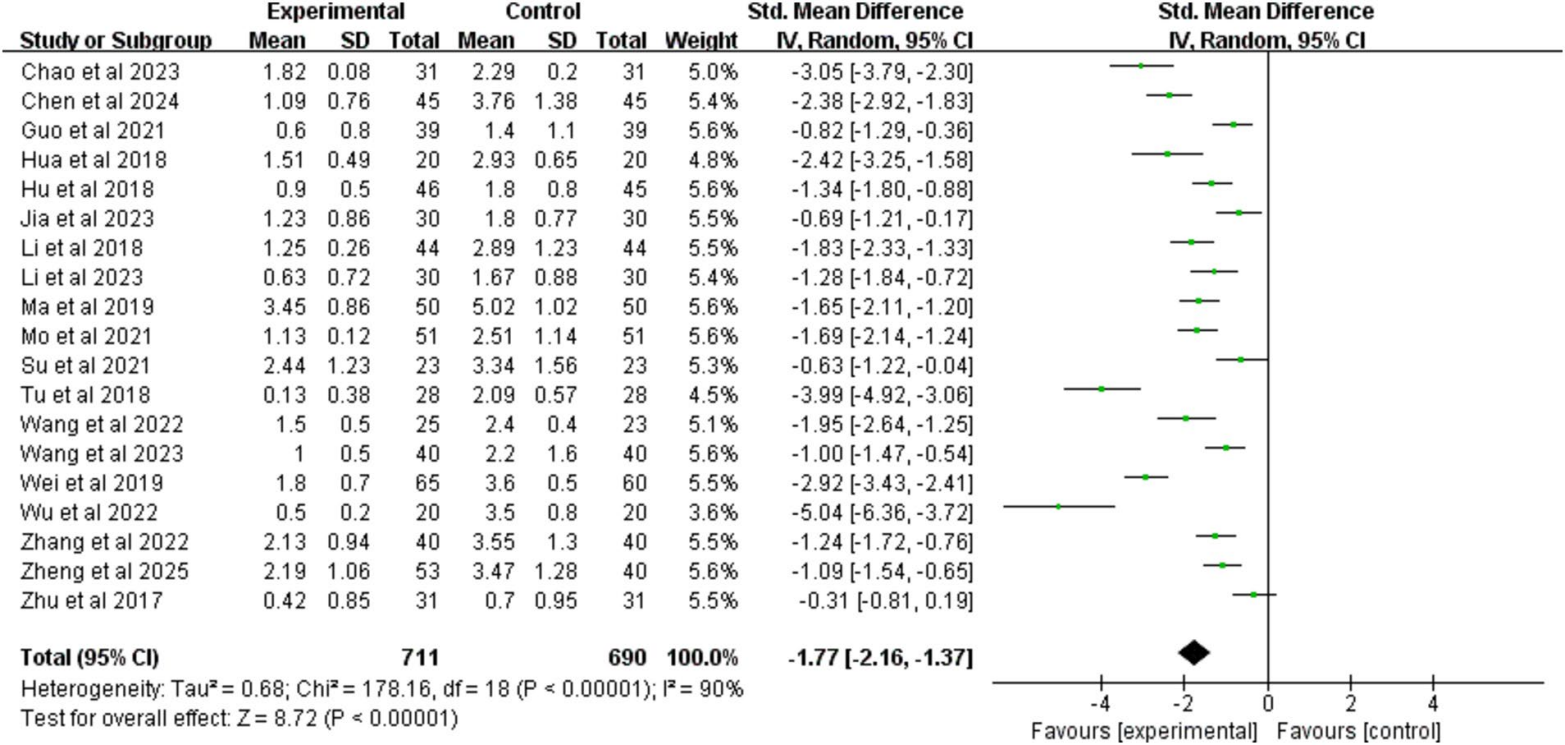
Forest plot of pain score at last follow-up.

**Figure 3:**
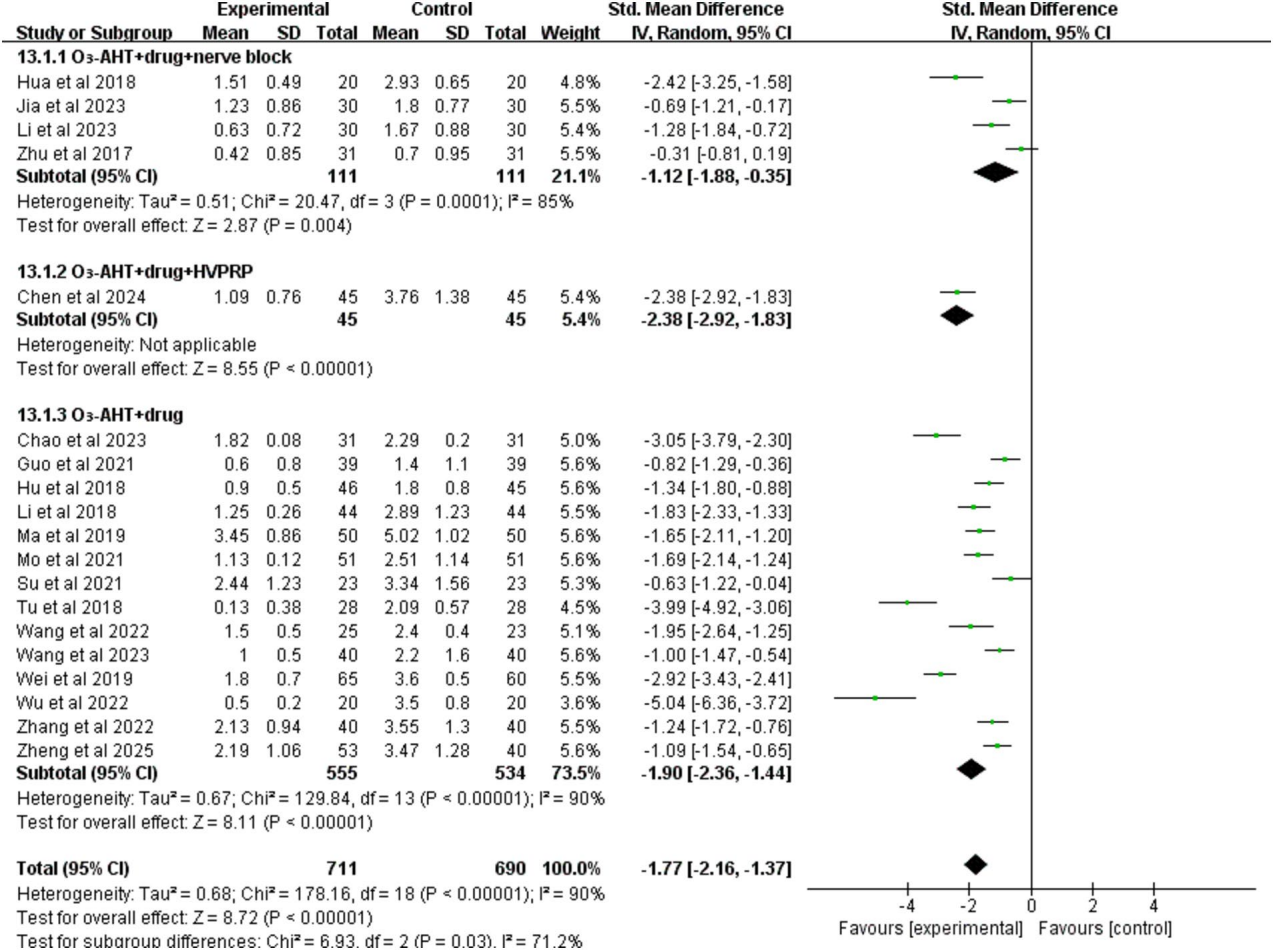
Forest diagram of pain scores for different interventions.

**Figure 4:**
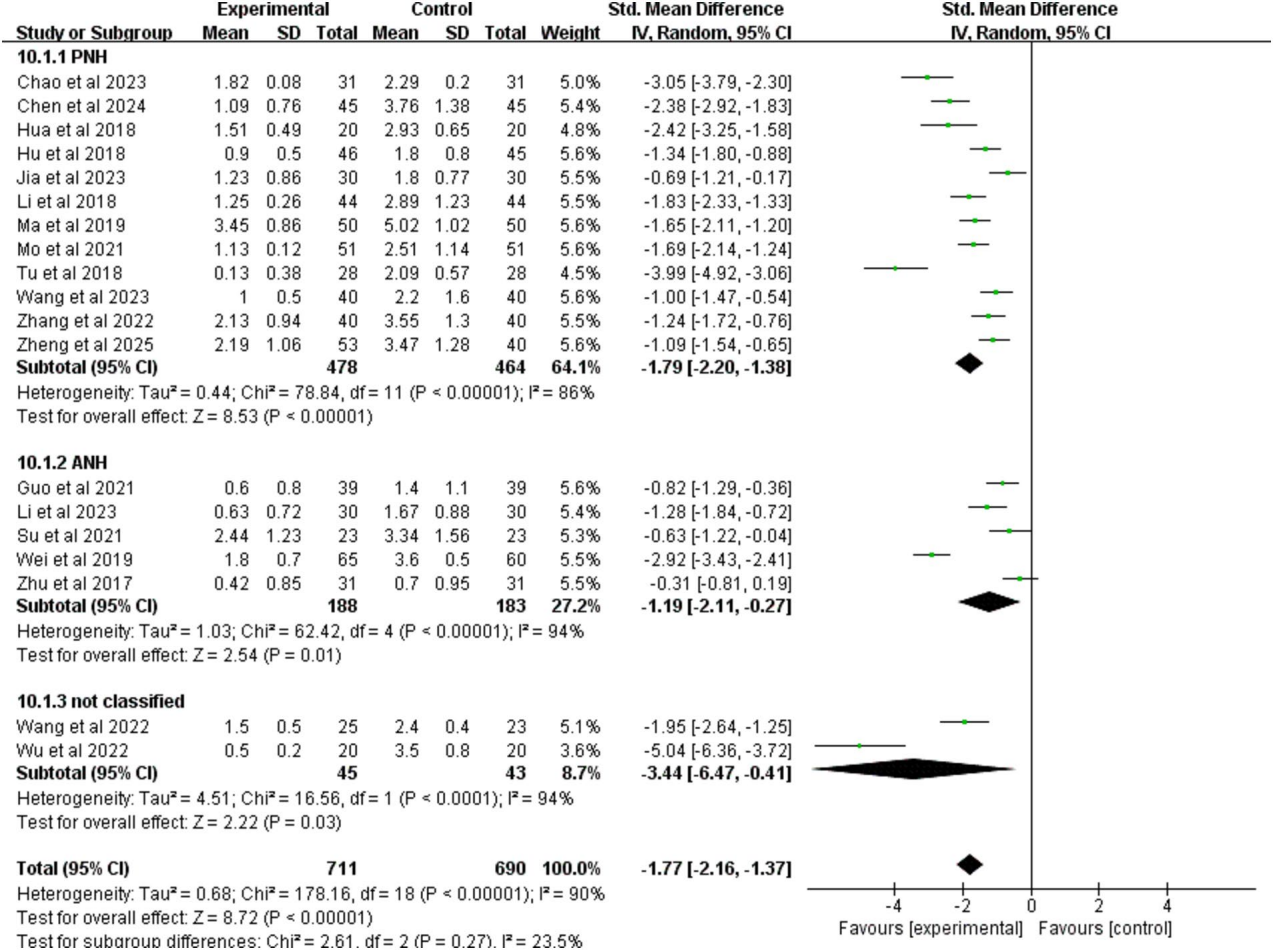
Forest plot of pain scores for different course of disease.

A pooled analysis of self-reported pain scores of patients at the end of follow-up in the 19 included studies, it was found that there was a significant improvement in pain (SMD = −1.77, 95% CI: −2.16 to −1.37, *p* < 0.01) (12–16, 18, 19, 26–37). However, substantial heterogeneity was observed among studies (I^2^ = 90%), indicating the need for cautious interpretation (Figure 2). To further explore the source of heterogeneity, we performed subgroup analyses of outcomes based on intervention type. The results showed that the patient’s self-reported pain score was significantly lower than that of the control group in each combination treatment strategy (Figure 3). Subgroup analysis of outcomes according to the duration of the disease showed that O_3_-OAH was equally effective for different course of disease (Figure 4).

Based on the number of studies included at each follow-up time point, we analyzed patient self-reported pain score at different time points. Post-treatment analysis was qualitative owing to the presence of only one study, showing that the analgesic effect of the O_3_-AHT group was significantly superior to that in the control group (*p* < 0.01). Quantitative analysis was used for the remaining follow-up time points, showing that compared to the control group, pain scores in the O_3_-AHT group were significantly reduced, and the quantitative outcome effects were as follows: Day 7 (MD = −1.28, 95% CI: −1.64 to −0.92, *p* < 0.01), Week 2 (MD = −1.16, 95% CI: −1.57 to −0.76, *p* < 0.01), Month 1 (MD = −1.25, 95% CI: −1.79 to −0.71, *p* < 0.01), Month 3 (MD = −1.42, 95% CI: −2.03 to −0.82, *p* < 0.01), and Month 6 (MD = −1.77, 95% CI: −3.51 to −0.04, *p* = 0.04) (Table 3).

**Table 3:** Outcome evaluation at each time point after treatment.

Outcome	Follow-up time	Number of studies	Heterogeneity		Model	Results	
			*p*	*I^2^* (%)		*MD*/*SMD/RR* (95% CI)	*p*-value
Pain score	After treatment	1	/	/	Random	−1.57 (−1.94 to −1.20)	< 0.01
	1 W	12	< 0.01	91	Random	−1.28 (−1.64 to − 0.92)	< 0.01
	2 W	3	0.10	57	Random	−1.16 (−1.57 to −0.76)	< 0.01
	1 M	9	< 0.01	97	Random	−1.25 (−1.79 to −0.71)	< 0.01
	3 M	6	< 0.01	95	Random	−1.42 (−2.03 to −0.82)	< 0.01
	6 M	2	< 0.01	98	Random	−1.77 (−3.51 to −0.04)	0.04
IL-6	After treatment	3	< 0.01	94	Random	−2.30 (−3.59 to −1.01)	< 0.01
	4D	1	/	/	Random	−0.67 (−1.19 to −0.15)	0.01
	7D	1	/	/	Random	−1.33 (−1.89 to −0.77)	< 0.01
	10D	2	0.66	0	Fixed	−0.68 (−1.07 to −0.29)	< 0.01
	20D	2	0.04	76	Random	−1.29 (−2.16 to −0.42)	< 0.01
	1 M	2	< 0.01	88	Random	−1.39 (−2.65 to −0.13)	0.03
IL-1β	4D	1	/	/	Random	−1.22 (−1.78 to −0.67)	< 0.01
	7D	2	< 0.01	89	Random	−1.83 (−3.02 to −0.64)	< 0.01
	2 W	1	/	/	Random	−4.15 (−4.85 to −3.45)	< 0.01
	1 M	1	/	/	Random	−3.10 (−3.68 to −2.52)	< 0.01
CD4^+^/CD8^+^	After treatment	1	/	/	Random	2.19 (1.74 to 2.63)	< 0.01
	4D	1	/	/	Random	0.65 (0.02 to 1.29)	0.04
	6D	1	/	/	Random	2.25 (1.44 to 3.06)	< 0.01
	8D	1	/	/	Random	0.39 (−0.24 to 1.01)	0.23
	10D	1	/	/	Random	0.56 (0.19 to 0.92)	< 0.01
	15D	1	/	/	Random	0.53 (−0.01 to 1.16)	0.10
	20D	1	/	/	Random	0.57 (0.20 to 0.93)	< 0.01
TNF-α	After treatment	3	< 0.01	98	Random	−0.83 (−3.11 to 1.45)	0.47
	7D	1	/	/	Random	−0.65 (−1.04 to 0.25)	< 0.01
	10D	2	0.14	54	Random	−0.88 (−1.48 to −0.29)	< 0.01
	2 W	1	/	/	Random	−0.80 (−1.20 to −0.40)	< 0.01
	20D	2	0.82	0	Fixed	−0.82 (−1.21 to −0.42)	< 0.01
	1 M	3	0.13	50	Fixed	−1.26 (−1.55 to −0.96)	< 0.01
PSQI	7D	1	/	/	Random	−4.07 (−4.76 to −3.38)	< 0.01
	10D	1	/	/	Random	−4.03 (−5.71 to −2.35)	< 0.01
	20D	1	/	/	Random	−2.74 (−4.17 to- 1.31)	< 0.01
	1 M	2	< 0.01	98	Random	−4.05 (−8.03 to −0.08)	0.05
	3 M	1	/	/	Random	−5.48 (−6.14 to −4.82)	< 0.01
	9 M	1	/	/	Random	−6.32 (−7.18 to 5.46)	< 0.01
Effective rate	After treatment	4	0.54	0	Fixed	1.28 (1.12 to 1.45)	< 0.01
	7D	1	/	/	Fixed	1.59 (0.73 to 3.46)	0.24
	1 M	1	/	/	Fixed	1.54 (1.18 to 2.02)	< 0.01
	3 M	1	/	/	Fixed	1.05 (0.92 to 1.20)	0.44
WHOQOL-BREF	7D	2	0.52	0	Fixed	0.38 (0.25 to 0.51)	< 0.01
	2 W	1	/	/	Random	0.8 (0.58 to 1.02)	< 0.01
	1 M	2	0.05	74	Random	0.76 (0.46 to 1.05)	< 0.01
	3 M	1	/	/	Random	0.60 (0.41 to 0.79)	< 0.01

#### 3.3.2 Pain relief effectiveness rate

**Figure 5:**
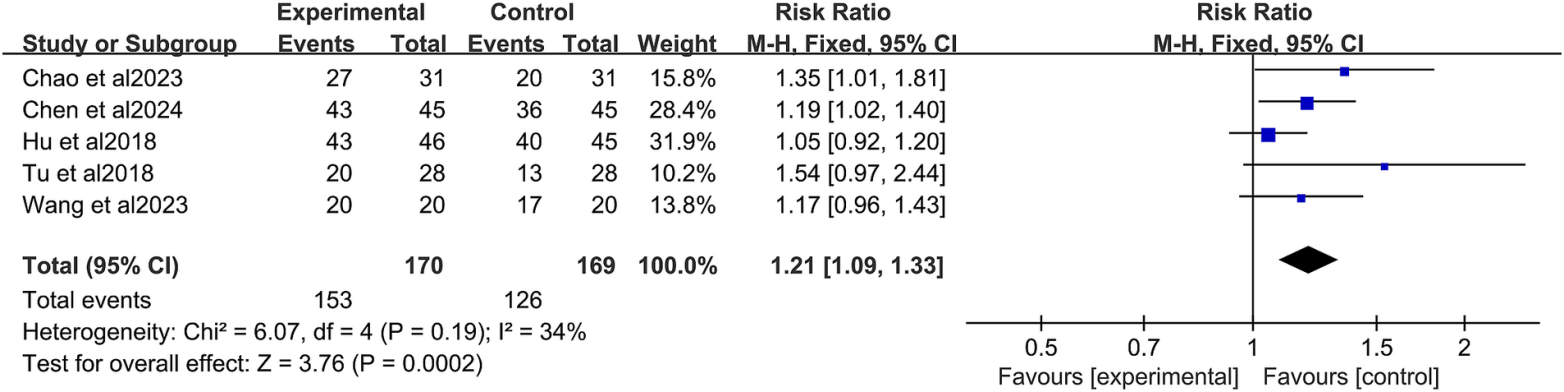
Forest diagram of the effective rate of pain relief at the last follow-up.

Five (12, 13, 19, 31, 32) studies reported the pain relief effectiveness rates (pain score decreased by > 50% or pain score < 5), involving 339 patients. Owing to low heterogeneity among studies (I^2^ = 34%, *p* = 0.19), a fixed-effects model was used for meta-analysis at the final follow-up, showing a significantly higher effectiveness rate in the O₃-AHT group (RR = 1.21, 95% CI: 1.09 to 1.33, *p* < 0.01) (Figure 5).

Based on the differences in the number of studies that could be included at different follow-up time points, we conducted a stratified analysis of the effectiveness rate of pain relief. Among them, data from four studies were available for pooling after treatment, and the meta-analysis results revealed that the effectiveness rate in the O_3_-AHT group was significantly higher than that in the control group (RR = 1.28, 95% CI: 1.12 to 1.45, *p* < 0.01). However, only one study provided relevant data at 7 days, 1 month, and 3 months; thus, qualitative analysis was adopted. The results of these individual studies consistently indicated that the effectiveness rate of the O_3_-AHT treatment group had substantial advantages over the control group.

#### 3.3.3 Levels of inflammatory factors and immune function indicators

**Figure 6:**
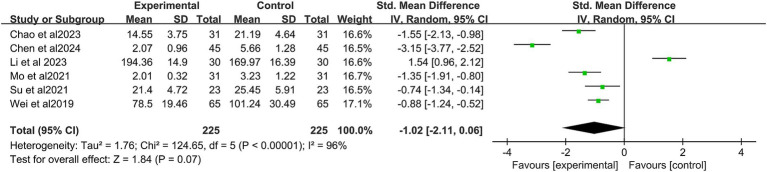
Forest diagram of TNF-*α* in peripheral blood at last follow-up.

**Figure 7:**
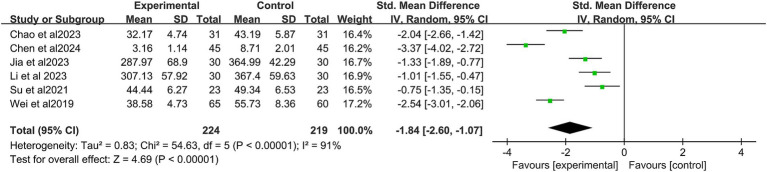
Forest diagram of IL-6 in peripheral blood at the last follow-up.

**Figure 8:**
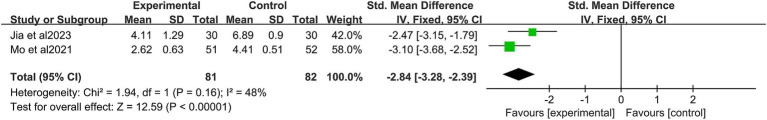
Forest diagram of IL-1β in peripheral blood at the last follow-up.

**Figure 9:**
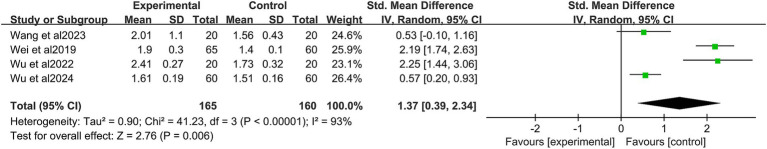
Forest plot of CD4^+^/CD8^+^ ratio in peripheral blood at last follow-up.

Ten studies reported the inflammatory factor levels and immune function indicators, of which 6 reported TNF-*α* levels (12, 13, 16, 29, 30, 36), 6 reported IL-6 levels (12, 13, 15, 16, 30, 36), 2 reported IL-1β levels (15, 29), and 4 reported immune function indicator CD4^+^/CD8^+^ levels (17, 18, 32, 36). Random-effects models were used for meta-analysis (fixed-effects model was used for IL-1β because of low heterogeneity), and the final follow-up results revealed that compared with the control group, patients in the O₃-AHT group exhibited significantly reduced levels of TNF-*α* (SMD = −1.02, 95% CI: −2.11 to 0.06, *p* < 0.01) (Figure 6), IL-6 (SMD = −1.84, 95% CI: −2.60 to −1.07, *p* < 0.01) (Figure 7), and IL-1β (SMD = −2.84, 95% CI: −3.28 to −2.39, *p* < 0.01) (Figure 8), while the CD4^+^/CD8^+^ ratio was significantly increased (SMD = 1.37, 95% CI: 0.39 to 2.34, *p* < 0.01) (Figure 9). These results suggest that O₃-AHT may alleviate ZAP through anti-inflammatory and immunomodulatory mechanisms.

The results of each follow-up time point were analyzed, and TNF-*α* was performed at 7 days and 2 weeks after treatment. IL-6 at 4 and 7 days after treatment; The results showed that the anti-inflammatory effect of the O3-AHT group was significantly better than that of the control group (*p* < 0.01), and the results showed that the inflammatory factors (IL-6, IL-1β) levels showed a significant downward trend during treatment, TNF-α showed an upward trend after treatment, and showed a significant downward trend in the rest of the time after treatment. The quantitative outcome effects were as follows:

TNF-α: post-treatment (SMD = −0.83, 95% CI: −3.11 to 1.45, *p* = 0.47), day 10 (SMD = −0.88, 95% CI: −1.48 to −0.29, *p* < 0.01), week 2 (SMD = −0.80, 95% CI: −1.20 to −0.40, *p* < 0.01), day 20 (SMD = −0.82, 95% CI: −1.21 to −0.42, *p* < 0.01), month 1 (SMD = −1.26, 95% CI: −1.55 to −0.96, *p* < 0.01). IL-6: post-treatment (SMD = −2.30, 95% CI: −3.59 to −1.01, *p* < 0.01), day 10 (SMD = −0.68, 95% CI: −1.07 to −0.29, *p* < 0.01), day 20 (SMD = −1.29, 95% CI: −2.16 to −0.42, *p* < 0.01), month 1 (SMD = −1.39, 95% CI: −2.65 to −0.13, *p* = 0.03). IL-1β: day 7 (SMD = −1.83, 95% CI: −3.02 to −0.64, *p* < 0.01) (Table 3).

Qualitative analysis showed that the CD4^+^/CD8^+^ ratio of patients in the O₃-AHT group showed an upward trend during treatment (except for the 8th day after treatment [*p* = 0.23]) compared with the control group, indicating improved immune function (Table 3).

#### 3.3.4 Quality of life score

**Figure 10:**
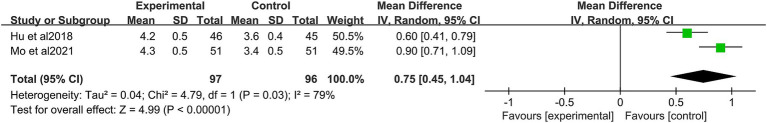
Forest diagram of quality of life at last follow-up.

Two studies reported quality of life scores (19, 29) using the WHOQOL-BREF scale. Owing to the high heterogeneity between studies (I^2^ = 79%, *p* = 0.03), a random-effects model was employed. Results revealed that the quality of life score in the O₃-AHT group was significantly higher than that in the control group (MD = 0.75, 95% CI: 0.45 to 1.04, *p* < 0.01) (Figure 10).

Based on the analysis of the results of each follow-up time point, there was only one study at 2 weeks and 3 months after treatment, so a qualitative analysis was used, and the results showed that the life treatment of the O_3_-AHT group was significantly better than that of the control group (*p* < 0.01). Quantitative analysis was used at the rest of the follow-up time points, and the WHOQOL-BREF score showed significant and continuous improvement. Quantitative outcome effects were as follows: day 7 (MD = 0.38, 95% CI: 0.25 to 0.51, *p* < 0.01), month 1 (MD = 0.76, 95% CI: 0.46 to 1.05, *p* < 0.01) (Table 3).

#### 3.3.5 Sleep quality

**Figure 11:**
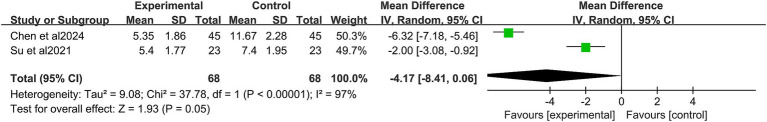
Forest diagram of sleep quality at last follow-up.

Two studies reported sleep quality (13, 30) using the PSQI scale. Owing to high heterogeneity between studies (I^2^ = 97%, *p* < 0.01), a random-effects model was employed. Results revealed that the sleep quality score in the O₃-AHT group was significantly higher than that in the control group (MD = −4.17, 95% CI: −8.41 to 0.06, *p* = 0.05) (Figure 11).

Based on the available data, we analysed the data at each follow-up time point: data from 2 studies at 1 month (MD = −4.05, 95% CI: −8.03 to −0.08, *p* = 0.05), while other time points reported by single studies were qualitatively described. Results revealed that the O₃-AHT group’s improvement effect on patient sleep quality demonstrated a positive trend of enhancement over time (Table 3).

#### 3.3.6 Adverse reactions

**Figure 12:**
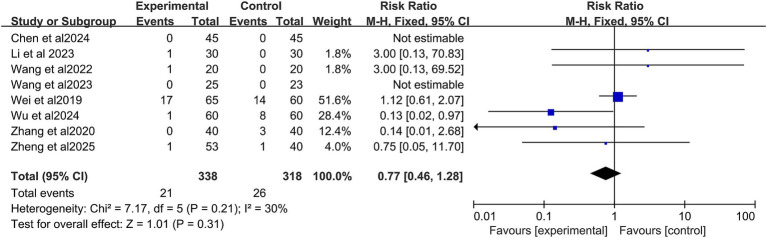
Forest diagram of adverse reactions.

Eight studies reported adverse events (13, 16, 17, 32–34, 36, 37), primarily including mild dizziness, nausea, and local discomfort. The overall incidence of adverse events in the O₃-AHT group was low (RR = 0.77, 95% CI: 0.46 to 1.28, *p* = 0.31), indicating good treatment safety in the O₃-AHT group (Figure 12).

### 3.4 Bias risk assessment

**Figure 13:**
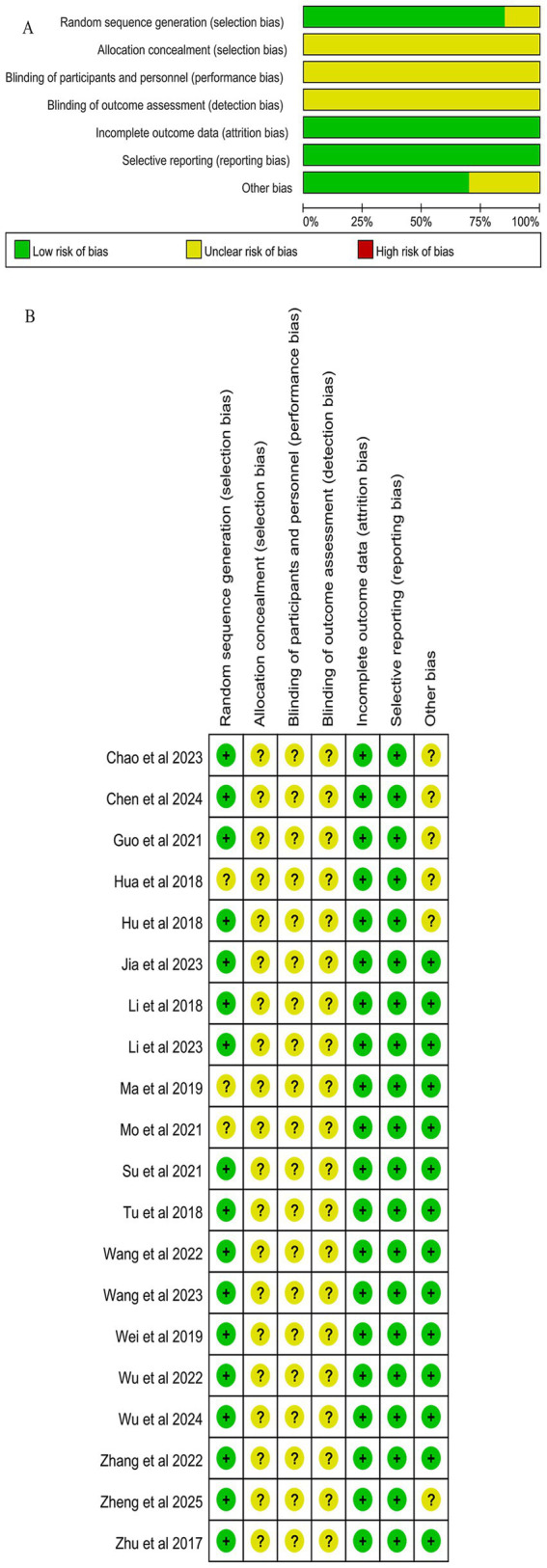
Risk of bias assessment for included studies: **(A)** Overall risk of bias. **(B)** Risk of bias characteristics for each included study.

The studies included in this meta-analysis were of acceptable quality in terms of randomization, with relatively complete data reporting. However, there may be risks in the core methodological aspects of allocation concealment and blinding, as the studies did not describe any allocation concealment measures, and owing to the invasive nature of the procedures, it was impossible to blind patients and operators, and the lack of blinding for assessors resulted in significant risk of bias in the assessment of subjective outcome indicators (such as pain scores). This constitutes the main limitation of this systematic review. Therefore, the strength of evidence regarding O₃-AHT significantly improving subjective experiences in patients with herpes zoster neuralgia is weakened, and further methodologically rigorous high-quality studies are warranted for confirmation (Figure 13).

### 3.5 Heterogeneity, sensitivity, and publication bias

For the primary outcome “patient-reported pain score”, meta-analyses showed a high degree of heterogeneity between studies (I^2^ = 90%). Although we performed subgroup analyses based on the type of intervention and the length of the course of the disease, heterogeneity was still high. All outcome sensitivity analyses showed relatively robust results. Egger’s test suggested that there may be publication bias in patients’ self-reported pain scores (*p* = 0.003), while no publication bias was found in the remaining results (Table 4).

**Table 4:** Publication bias.

Methods	Pain score	Sleep quality	remission rate	Adverse events	Inflammatory factor				Living quality
					TNF-α	IL-6	CD4+/CD8+	IL-1β	
Egger’s test	0.003	/	0.603	0.057	0.831	0.953	0.643	/	/
Begg’s test	0.039	0.317	0.851	0.348	0.573	0.573	0.497	0.317	0.317
Begg’s test (continuity corrected)	0.042	1.000	1.000	0.452	0.707	0.707	0.734	1.000	1.000

### 3.6 Trial sequential analysis

**Figure 14:**
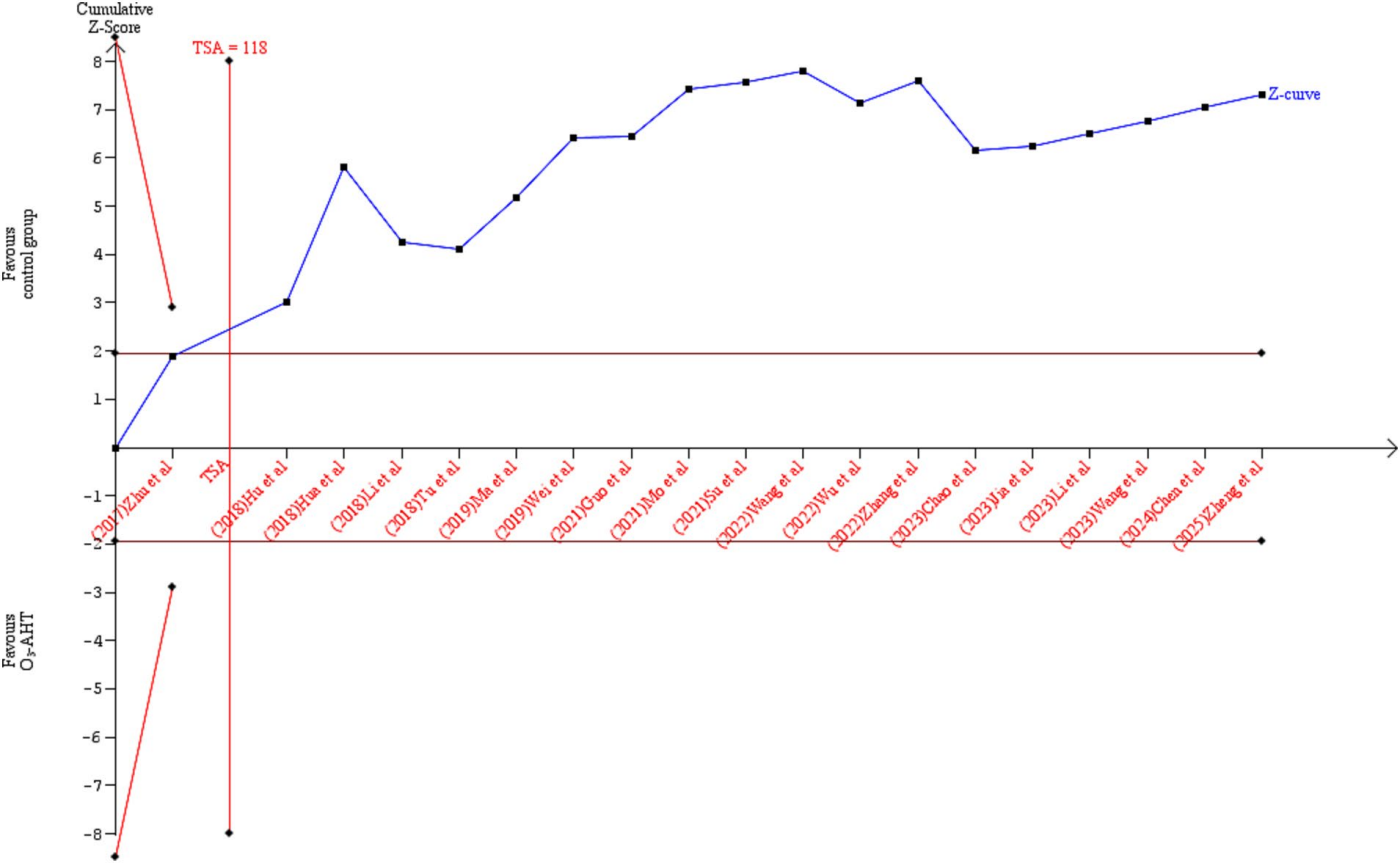
Trial sequential analysis of pain scores.

The TSA for the primary outcome “patient-reported pain score” showed that the cumulative Z-curve crossed the TSA monitoring boundary. And the current accumulated sample size has exceeded the amount of information required (Figure 14).

### 3.7 Quality of evidence

The quality of the evidence for patient-reported pain scores was ‘very low’ and ‘low’ to ‘moderate’ for other outcomes (Table 5).

**Table 5:** Overall quality of evidence assessment based on the Grading of Recommendations Assessment, Development and Evaluation (GRADE) scoring guidelines.

Outcomes	Illustrative comparative risks* (95% CI)		Relative effect	No of Participants	Quality of the evidence	Comments
	Assumed risk	Corresponding risk	(95% CI)	(studies)	(GRADE)	
	Control	O3-AHT				
Pain score	/	1.32 lower (1.68 to 0.95 lower)	/	1,301	⨁◯◯◯	/
				(18 studies)	very low 1, 2, 3	
	
Pain relief rate	Study population		RR 1.21 (1.09 to 1.33)	339 (5 studies)	⨁⨁◯◯ low 1, 2	/
	746 per 1,000	902 per 1,000				
		(813 to 992)				
	Moderate					
	800 per 1,000	968 per 1,000				
		(872 to 1,000)				
TNF-α	/	1.02 standard deviations lower (2.11 lower to 0.07 higher)	/	445	⨁◯◯◯	SMD -1.02 (−2.11 to 0.07)
				(6 studies)	very low 1, 2	
	
IL-6	/	1.84 standard deviations lower (2.6 to 1.07 lower)	/	443	⨁◯◯◯	SMD -1.84 (−2.6 to −1.07)
				(6 studies)	very low 1, 2	
	
IL-1β	/	2.84 standard deviations lower (3.28 to 2.39 lower)	/	163	⨁⨁⨁◯	SMD -2.84 (−3.28 to −2.39)
				(2 studies)	moderate 1	
	
CD4+/CD8+	/	1.37 standard deviations higher (0.39 to 2.34 higher)	/	325	⨁◯◯◯	SMD 1.37 (0.39 to 2.34)
				(4 studies)	very low 1, 2, 3	
	
Quality of life	/	0.75 higher (0.45 to 1.04 higher)	/	193	⨁◯◯◯	/
				(2 studies)	very low 1, 2	
	
Sleep quality	/	4.17 lower (8.41 lower to 0.06 higher)	/	136	⨁◯◯◯	/
				(2 studies)	very low 1, 2	
	
Adverse events	Study population		RR 0.77 (0.46 to 1.28)	656	⨁⨁◯◯	/
	82 per 1,000	63 per 1,000		(8 studies)	low 1, 2	
		(38 to 105)				
	Moderate					
	13 per 1,000	10 per 1,000				
		(6 to 17)				

^*^The basis for the assumed risk (e.g., the median control group risk across studies) is provided in footnotes. The corresponding risk (and its 95% confidence interval) is based on the assumed risk in the comparison group and the relative effect of the intervention (and its 95% CI). CI: Confidence interval.

GRADE Working Group grades of evidence. High quality: Further research is very unlikely to change our confidence in the estimate of effect. Moderate quality: Further research is likely to have an important impact on our confidence in the estimate of effect and may change the estimate. Low quality: Further research is very likely to have an important impact on our confidence in the estimate of effect and is likely to change the estimate. Very low quality: We are very uncertain about the estimate.

1 Included studies have considerable bias in randomization, allocation concealment, and blinding. 2 Wide confidence interval. 3 I^2^ ≥ 75%.

## 4 Discussion

This study comprehensively synthesized data from 20 RCT studies involving 1,519 patients to conduct a systematic evaluation of the efficacy and safety of O₃-AHT in treating ZAP. Meta-analysis results revealed that ozone therapy significantly reduced patients’ self-reported pain scores (SMD = −1.77), improved pain relief effectiveness (RR = 1.21), decreased inflammatory factor levels (TNF-*α* SMD = −1.02; IL-6 SMD = −1.84), and improved quality of life (MD = 0.75), without increasing the risk of adverse reactions. These findings provide evidence supporting the potential application of O₃-AHT in ZAP management. For patients with ZAP who respond poorly to or cannot tolerate conventional pharmacological therapy, O_3_-AHT may serve as an effective complementary or alternative treatment option.

O₃-AHT may exert analgesic effects through multiple mechanisms (38–40). Basic research indicates that ozone can alleviate pain perception by inhibiting the release of pain-related inflammatory factors (such as serotonin and bradykinin) in peripheral tissues (41, 42). In addition, ozone can improve local tissue metabolism, relieve hypoxic conditions at nerve endings, and promote nerve repair (43). Clinical studies have also found that ozone therapy can indirectly alleviate pain by improving sleep quality through elevation of serum brain-derived neurotrophic factor and *γ*-aminobutyric acid levels (44). Consistent with these mechanisms, this study found that O₃-AHT treatment significantly reduced the inflammatory factor levels, further supporting the role of its anti-inflammatory mechanism in mediating analgesia. It is worth noting that recent studies have indicated that ozone therapy may have an emotional regulation effect (45, 46). This may primarily work by modulating oxidative stress, thereby enhancing metabotropic glutamate signal transmission, to improve patients’ depressive symptoms. Given the close bidirectional influence between emotional disorders (such as depression, anxiety) and neuropathic pain (47), ozone may indirectly enhance pain relief effects by improving patients’ emotional state, providing a new perspective for understanding its multidimensional efficacy.

We found that O₃-AHT resulted in an average reduction in patients’ self-reported pain scores by about 1.77 standardized units overall, with confidence intervals showing reductions between 1.37 and 2.16. This was higher than our pre-qualified clinical minimum difference (decrease ≥ 1.5 points or ≥ 30%) (24, 25). It suggests that O₃-AHT has clear clinical significance for most patients with ZAP, which translates into perceptible pain relief and functional improvement. In addition, the results of TSA showed that the cumulative Z-curve had crossed the monitoring boundary, and the current sample size far exceeded the required amount of information (RIS), which further confirmed that the analgesic effect was relatively robust and reliable.

Subgroup analysis showed that the effects of O₃-AHT combined with different treatment modalities were different, and the heterogeneity between different studies in each subgroup was still large。 This may stem from differences in ozone concentrations, course of treatment, or combination therapy across studies. O₃-AHT combined with pulsed radiofrequency has the best effect (SMD = −2.38), which may be attributed to its systemic regulatory effects, enabling simultaneous immunomodulatory and anti-inflammatory effects and the local effect of pulsed radiofrequency exerts a stronger analgesic synergistic effect. Compared with compound nerve block, pulsed radiofrequency has a more stable and long-lasting effect (48). Some studies have reported that HVPRF combined with ozone therapy is superior to ozone therapy alone (48), and that ultrasound-guided spinal nerve root PRF combined with ozone therapy shows significant efficacy in patients with ZAP across different disease durations (49); however, therapeutic efficacy appears to decline with prolonged disease duration (50). Moreover, spinal cord stimulation is becoming increasingly prevalent in ZAP clinical practice (51), considering that the use of anticoagulants in O₃-AHT may limit the combination of O₃-AHT and electrical spinal cord stimulation to a certain extent. This study also found that O₃-AHT significantly improved patients’ quality of life, with pain relief and sleep improvement (13, 30) likely being important reasons for the enhanced quality of life (19, 29). Additionally, it is important to note that all of the studies we included combined O₃-AHT on top of other treatments (pharmacotherapy, nerve blocks, and pulsed radiofrequency), so we need to pay attention to the impact of background treatment regimens on outcomes in terms of safety analysis. Conventional drug treatments such as gabapentin and pregabalin often cause drowsiness, vertigo, peripheral edema, etc. (52–54). Nerve blocks may cause transient hypotension, slight discomfort at the puncture site, infection, and nerve damage (55, 56). Pulsed radiofrequency may cause skin numbness, pain, bleeding, infection, etc. (57). In contrast, the adverse reactions of O3-AHT are different, and most of them are short-lived and mild (58). Meta-analyses showed that compounding O3-AHT did not increase the additional burden on patients compared with other treatments in the control group. However, it should be noted that there have been rare case reports suggesting that ozone therapy may have serious risks (59) such as air plugs. Therefore, although this study shows a good safety of O3-AHT, its risk–benefit ratio still needs to be considered in clinical practice based on the specific situation of the patient and alternative treatment options (60, 61). However, this study has the following limitations. First, the methodological quality of the included studies varied considerably, with some studies failing to provide detailed descriptions of random sequence generation and allocation concealment methods, which may affect the reliability of the results. Second, substantial heterogeneity existed among studies; although subgroup analyses reduced some heterogeneity, there were still residual control factors (such as ozone concentration, treatment frequency, treatment course, etc.) that may affect the stability of the results. Third, the included studies were primarily published in Chinese, which may introduce language and publication bias. Fourth, although TSA analysis suggests that the outcomes are relatively robust and reliable, they are limited by the high heterogeneity, potential bias, and relatively small trials of the included studies, and the GRADE evidence evaluation suggests that the results are mostly of very low to moderate quality, so the results still need to be viewed conservatively. Finally, long-term follow-up data were limited, preventing assessment of the long-term efficacy and sustainability of O₃-AHT.

Based on the above results, we propose the following recommendations for clinical practice and research. Clinically, ozone therapy, especially O₃-AHT combined multimodal treatment regimen, may be considered as part of a comprehensive management strategy for herpes zoster-associated pain. From a research perspective, future studies should include large-sample, multicenter, high-quality RCTs are needed in the future to explore more standard treatment options and use unified efficacy evaluation criteria to standardized outcome measures, long-term follow-up, and cost-effectiveness analyses, as well as further exploration of the biological mechanisms underlying ozone therapy.

## 5 Conclusion

This meta-analysis confirms that O₃-AHT may be effectively alleviate ZAP, improve inflammatory markers and quality of life, regulating mood, and demonstrates a good safety profile. The therapeutic effect of O₃-AHT combined with other treatments appears superior to that of O₃-AHT monotherapy. However, owing to limitations in the number and quality of included studies, these conclusions should be interpreted with caution and require validation via further high-quality research. Clinicians are advised to use O₃-AHT judiciously in the comprehensive management of ZAP based on individual patient characteristics and available medical resources.

## Data Availability

The original contributions presented in the study are included in the article/supplementary material, further inquiries can be directed to the corresponding author.

## References

[1] Johnson RW Rice ASC. Clinical practice. Postherpetic neuralgia. N Engl J Med. (2014) 371:1526–33. doi: 10.1056/NEJMcp1403062, 25317872

[2] Forbes HJ Thomas SL Smeeth L Clayton T Farmer R Bhaskaran K . A systematic review and meta-analysis of risk factors for postherpetic neuralgia. Pain. (2016) 157:30–54. doi: 10.1097/j.pain.0000000000000307, 26218719 PMC4685754

[3] Kawai K Gebremeskel BG Acosta CJ. Systematic review of incidence and complications of herpes zoster: towards a global perspective. BMJ Open. (2014) 4:e004833. doi: 10.1136/bmjopen-2014-004833, 24916088 PMC4067812

[4] Cohen SP Mao J. Neuropathic pain: mechanisms and their clinical implications. BMJ. (2014) 348:f7656. doi: 10.1136/bmj.f7656, 24500412

[5] Conference TPC. The Polyanalgesic consensus Conference (PACC): recommendations on intrathecal drug infusion systems best practices and guidelines. Neuromodulation. (2017) 20:405–6. doi: 10.1111/ner.12618, 28593641

[6] Finnerup NB Kuner R Jensen TS. Neuropathic pain: from mechanisms to treatment. Physiol Rev. (2021) 101:259–301. doi: 10.1152/physrev.00045.2019, 32584191

[7] Smith NL Wilson AL Gandhi J Vatsia S Khan SA. Ozone therapy: an overview of pharmacodynamics, current research, and clinical utility. Med Gas Res. (2017) 7:212–9. doi: 10.4103/2045-9912.215752, 29152215 PMC5674660

[8] Elvis AM Ekta JS. Ozone therapy: a clinical review. J Nat Sci Biol Med. (2011) 2:66–70. doi: 10.4103/0976-9668.82319, 22470237 PMC3312702

[9] Liu Y Shen T Li Q Yu X Liu Y Zhou C . Various gases for the treatment of neuropathic pain: mechanisms, current status, and future perspectives. Med Gas Res. (2025) 15:488–95. doi: 10.4103/mgr.MEDGASRES-D-24-00161, 40300884 PMC12124698

[10] Bocci V Zanardi I Travagli V. Ozone: a new therapeutic agent in vascular diseases. Am J Cardiovasc Drugs. (2011) 11:73–82. doi: 10.2165/11539890-000000000-00000, 21446774

[11] Chen N Feng L Li M Bai F Yang K Du W . Systematic review of the efficacy of ozone autologous blood transfusion therapy in the treatment of postherpetic neuralgia. Chinese Med Herald. (2021) 23:641–6.

[12] Cao ZP Wang L He JL. Clinical efficacy of ozone autohemotherapy in the treatment of postherpetic neuralgia. Contemp Med. (2023) 29:108–11.

[13] Chen JX Li YQ Yi QX Yuan T Xie ZH. Clinical study of high voltage pulsed radio frequency combined with ozone autohemotherapy for postherpetic neuralgia. Health Med Res Pract. (2024) 21:90–4. doi: 10.11986/j.issn.1673-873X.2024.05.16

[14] Guo YY Xue ZX Duan LZ Chen JP Wang Q Sun LH . Therapeutic effect of medical ozone autohemotherapy on acute herpes zoster. Chin J Pain Med. (2021) 27:438–42. doi: 10.3969/j.issn.1006-9852.2021.06.007

[15] Jia CQ Wang YX Wu X Zheng K Xue MH. Efficacy of ozone autohemotherapy for postherpetic neuralgia and its effects on IL-1β, IL-6, and β-endorphin. J Shandong First Med Univ Shandong Academy Medical Science. (2023) 44:352–5. doi: 10.3969/j.issn.2097-0005.2023.05.006

[16] Liyan L Yanxiu W Xue W Kai Z Minhua X. Clinical efficacy of ozone autohemotherapy combined with conventional therapy in treating acute-phase herpes zoster pain and its effects on related cytokines. J Shandong First Med Univ Shandong Academy Med Sci. (2023) 44:576–80. doi: 10.3969/j.issn.2097-0005.2023.08.004

[17] Xinjun W Sheng L Wei Z Xiaoli H. Spinal nerve radio frequency combined with immune ozone autohemotherapy for herpes zoster-related pain. Chin Med Innov. (2024) 21:28–32.

[18] Wu X Wang YX Zhu LL Cao LM Yang G Zheng K . Effect of ozone autohemotherapy on acute herpes zoster and its influence on T lymphocyte subsets. Chin J Pain Med. (2022) 18:216–21. doi: 10.3760/cma.j.cn101658-20200719-00132

[19] Hu B Zheng J Liu Q Yang Y Zhang Y. The effect and safety of ozone autohemotherapy combined with pharmacological therapy in postherpetic neuralgia. J Pain Res. (2018) 11:1637–43. doi: 10.2147/JPR.S154154, 30214273 PMC6118245

[20] Luo YX Zhang KB Li XZ. Clinical observation of pulsed radio frequency combined with ozone therapy for thoracic postherpetic neuralgia. Chin J Pain Med. (2018) 24:314–8.

[21] Zhang SB Yang XL Huang H Mei DC Wang YX. Clinical report of 40 cases of postherpetic neuralgia treated with medical ozone autohemotherapy. Chin J Pain Med. (2016) 22:716–8.

[22] Shamseer L Moher D Clarke M Ghersi D Liberati A Petticrew M . Preferred reporting items for systematic review and meta-analysis protocols (PRISMA-P) 2015: elaboration and explanation. BMJ. (2015) 350:g7647. doi: 10.1136/bmj.g7647 25555855

[23] Liberati A Altman DG Tetzlaff J Mulrow C Gotzsche PC Ioannidis JPA . The PRISMA statement for reporting systematic reviews and meta-analyses of studies that evaluate healthcare interventions: explanation and elaboration. BMJ. (2009) 339:b2700. doi: 10.1136/bmj.b2700, 19622552 PMC2714672

[24] Farrar JT Young JP LaMoreaux L Werth JL Poole MR. Clinical importance of changes in chronic pain intensity measured on an 11-point numerical pain rating scale. Pain. (2001) 94:149–58. doi: 10.1016/S0304-3959(01)00349-9, 11690728

[25] Ostelo RWJG Deyo RA Stratford P Waddell G Croft P Von Korff M . Interpreting change scores for pain and functional status in low back pain: towards international consensus regarding minimal important change. Spine (Phila Pa 1976). (2008) 33:90–4. doi: 10.1097/BRS.0b013e31815e3a10, 18165753

[26] Hua L Ou CH Zhao JM. Study on ozone major autohemotherapy combined with nerve block therapy for postherpetic neuralgia. Sichuan Med J. (2018) 39:555–8. doi: 10.16252/j.cnki.issn1004-0501-2018.05.020

[27] Li SR Chen CR Yang J Tian F Lu Y Wei R . Effect of immune ozone autohemotherapy on the incidence of postherpetic neuralgia in elderly patients. Hebei Med J. (2018) 40:2135–8.

[28] Rui M Xiufen K. Analysis of the Effect of Ozone Autohemotherapy on Clinical Efficacy and Quality of Life in Patients with Postherpetic Neuralgia. Int J Nurs. (2019) 38:2374–8. doi: 10.3760/cma.j.issn.1673-4351.2019.15.023

[29] Zhiwei M Dongyang L Han D Xue L Wusheng H Jiabiao P . Efficacy evaluation of ozone autohemotherapy combined with drug therapy for postherpetic neuralgia. J Diagn Ther Dermato-venereology. (2021) 28:382–5.

[30] Jianlin S Shiyao W. Clinical efficacy of platelet-rich plasma combined with ozone major autohemotherapy in treating acute-phase herpes zoster and its effects on inflammatory factors. Guangdong Med J. (2021) 42:444–8. doi: 10.13820/j.cnki.gdyx.20203843

[31] Tu T Wang J Dong SH. Clinical observation of ultrasound-guided nerve block combined with autologous blood ozone therapy in the treatment of postherpetic neuralgia. China Digit Med. (2018) 13:13–5.

[32] Wang L Shi HC Gao LQ Xi LL Ren YE Liu GZ. Short-term efficacy of ozone autohemotherapy combined with conventional drug therapy in patients with postherpetic neuralgia and its effect on cellular immune function mediated by T lymphocyte subsets. Chin J Pain Med. (2023) 19:601–5. doi: 10.3760/cma.j.cn101658-20200830-00152

[33] Wang X Yao TP Han CF Yang WQ He JD Guo YY . Optimization effect of ozone autohemotherapy on oral medication treatment in elderly female patients with herpetic neuralgia. Chin J Pain Med. (2022) 18:210–5. doi: 10.3760/cma.j.cn101658-20200517-00097

[34] Shuo Z. Observation on the efficacy of immune ozone autohemotherapy in the treatment of postherpetic neuralgia. [master’s thesis]. [Jilin Province]: Changchun Univ Chinese Med. (2020). doi: 10.26980/d.cnki.gcczc.2020.000516

[35] Yongqiang Z Peng Y. Observation on the efficacy of paravertebral block combined with ozone autohemotherapy for elderly herpes zoster. Chin J Pain Med. (2017) 23:272–6.

[36] Huaixiang W. Clinical Observation of Acyclovir Combined with Ozone Autohemorrhage in the Treatment of Herpes Zoster. [master’s thesis]. [Hubei Province]: China Three Gorges University. (2019). doi: 10.27270/d.cnki.gsxau.2019.000593

[37] Zheng C Xie Y Li H Zhang B Chen S Han W . Effectiveness and safety of stellate ganglion block with trioxygen autologous blood retransfusion therapy for facial postherpetic neuralgia in elderly patients. Sci Rep. (2025) 15:8025. doi: 10.1038/s41598-025-91847-7, 40055398 PMC11889258

[38] Ciborowski M Lipska A Godzien J Ferrarini A Korsak J Radziwon P . Combination of LC-MS- and GC-MS-based metabolomics to study the effect of ozonated autohemotherapy on human blood. J Proteome Res. (2012) 11:6231–41. doi: 10.1021/pr3008946, 23148940

[39] Pecorelli A Bocci V Acquaviva A Belmonte G Gardi C Virgili F . NRF2 activation is involved in ozonated human serum upregulation of HO-1 in endothelial cells. Toxicol Appl Pharmacol. (2013) 267:30–40. doi: 10.1016/j.taap.2012.12.001, 23253326

[40] Sancak EB Turkön H Çukur S Erimsah S Akbas A Gulpinar MT . Major ozonated autohemotherapy preconditioning ameliorates kidney ischemia-reperfusion injury. Inflammation. (2016) 39:209–17. doi: 10.1007/s10753-015-0240-z, 26282390

[41] Rowen RJ Robins H. Ozone therapy for complex regional pain syndrome: review and case report. Curr Pain Headache Rep. (2019) 23:41. doi: 10.1007/s11916-019-0776-y, 31062104 PMC6502773

[42] Kharrat A Elmounedi N Tmar MA Bahloul W Guidara AR Lajmi A . Effectiveness of ozone nucleolysis in alleviating pain and enhancing function in lumbar sciatica due to disc herniation: a minimally invasive approach. Clin Rheumatol. (2025) 44:475–85. doi: 10.1007/s10067-024-07255-1, 39627478

[43] Rivas-Arancibia S Guevara-Guzmán R López-Vidal Y Rodríguez-Martínez E Zanardo-Gomes M Angoa-Pérez M . Oxidative stress caused by ozone exposure induces loss of brain repair in the hippocampus of adult rats. Toxicol Sci. (2010) 113:187–97. doi: 10.1093/toxsci/kfp252, 19833740

[44] Li Y Feng X Ren H Huang H Wang Y Yu S. Low-dose ozone therapy improves sleep quality in patients with insomnia and coronary heart disease by elevating serum BDNF and GABA. Bull Exp Biol Med. (2021) 170:493–8. doi: 10.1007/s10517-021-05095-6, 33713235

[45] Sun W Xu L Liu Y. Research progress on the mechanism of ozone therapy in treating nervous system diseases. Medical Corps Armed Police Force. (2022) 33:907–10. doi: 10.14010/j.cnki.wjyx.2022.10.003

[46] Romanello D Martinelli M. A theoretical model for ozone therapy in depression treatment: enhancing metabotropic glutamate signaling through controlled oxidative stress. Med Gas Res. (2025) 15:210–1. doi: 10.4103/mgr.MEDGASRES-D-24-00123, 40070194 PMC11918475

[47] Vieira WF Coelho DRA Litwiler ST McEachern KM Clancy JA Morales-Quezada L . Neuropathic pain, mood, and stress-related disorders: a literature review of comorbidity and co-pathogenesis. Neurosci Biobehav Rev. (2024) 161:105673. doi: 10.1016/j.neubiorev.2024.105673, 38614452

[48] Kim ED Lee YI Park HJ. Comparison of efficacy of continuous epidural block and pulsed radiofrequency to the dorsal root ganglion for management of pain persisting beyond the acute phase of herpes zoster. PLoS One. (2017) 12:e0183559. doi: 10.1371/journal.pone.0183559, 28827823 PMC5565119

[49] Li XM Zhao QN Zhang JF . Ultrasound-guided high-voltage pulsed radio frequency combined with ozone injection for postherpetic neuralgia. Chin J Pain Med. (2023) 29:346–52.

[50] Ding XT Huang JZ Shen QS Wang RY Kan HM. Effects of pulsed radiofrequency combined with ozone on zoster-associated pain: a systematic review and meta-analysis. Med Gas Res. (2026) 16:76–81. doi: 10.4103/mgr.MEDGASRES-D-24-00150, 40580192 PMC12318573

[51] Li JL Qiao LBT Su B . Clinical study of spinal nerve root pulsed radio frequency combined with ozone therapy for postherpetic neuralgia. Health Med Res Pract. (2024) 21:47–50.

[52] Guan Y Ding X Cheng Y Fan D Tan L Wang Y . Efficacy of pregabalin for peripheral neuropathic pain: results of an 8-week, flexible-dose, double-blind, placebo-controlled study conducted in China. Clin Ther. (2011) 33:159–66. doi: 10.1016/j.clinthera.2011.02.007, 21444113

[53] Wang J Zhu Y. Different doses of gabapentin formulations for postherpetic neuralgia: a systematical review and meta-analysis of randomized controlled trials. J Dermatolog Treat. (2017) 28:65–77. doi: 10.3109/09546634.2016.1163315, 27798973

[54] Parsons B Pan X Xie L Chen Y Ortiz M Whalen E. Comparison of the efficacy and safety of pregabalin for postherpetic neuralgia in Chinese and international patients. J Pain Res. (2018) 11:1699–708. doi: 10.2147/JPR.S157856, 30214280 PMC6126478

[55] Lin ZM Wang HF Zhang F Ma JH Yan N Liu XF. The effect of erector spinae plane blockade on prevention of postherpetic neuralgia in elderly patients: a randomized double-blind placebo-controlled trial. Pain Physician. (2021) 24:E1109–18. 34704720

[56] Lu W He S Liu Q Gu Y Bai J. Comparative effectiveness of nerve block strategies for preventing postherpetic neuralgia in thoracic herpes zoster: a network meta-analysis. Front Neurol. (2025) 16:1612871. doi: 10.3389/fneur.2025.1612871, 40901665 PMC12399379

[57] Rui M Ni H Xie K Xu L Yao M. Progress in radiofrequency therapy for zoster-associated pain about parameters, modes, targets, and combined therapy: a narrative review. Pain Ther. (2024) 13:23–32. doi: 10.1007/s40122-023-00561-7, 37962817 PMC10796860

[58] Wang Y Qian X. Expert consensus on tri-oxygen autologous blood therapy. J Transl Med. (2018) 7:326–8.

[59] Shamohammadi M Mazraeh M Tayebi A Olamaeian F Moallem HH. Ozone therapy-associated pneumoperitoneum in a patient with low back pain: a case report. Int J Surg Case Rep. (2025) 130:111289. doi: 10.1016/j.ijscr.2025.111289, 40233642 PMC12019194

[60] Liu Z Weng Y Liu F Jiang D Wu C Chen Y . Efficacy and safety of short-term spinal cord stimulation and pulsed radiofrequency in the treatment of postherpetic neuralgia: a meta-analysis. Front Neurol. (2025) 16:1586995. doi: 10.3389/fneur.2025.1586995, 40606142 PMC12213773

[61] Liu Y Ming Z Jia S Su Y Wu K Yan W . Multi-Omics Insights into the Gut-Spinal Cord Axis: Paeoniflorin Mitigates Diabetic Neuropathic Pain in Rats through Microbial-Metabolic Crosstalk. Food Sci. Hum. Wellness. (2025). doi: 10.26599/FSHW.2025.9250837, 40606142

